# Impact of Low Dose Prenatal Ethanol Exposure on Glucose Homeostasis in Sprague-Dawley Rats Aged up to Eight Months

**DOI:** 10.1371/journal.pone.0059718

**Published:** 2013-03-22

**Authors:** Megan E. Probyn, Kylie R. Parsonson, Emelie M. Gårdebjer, Leigh C. Ward, Mary E. Wlodek, Stephen T. Anderson, Karen M. Moritz

**Affiliations:** 1 School of Biomedical Sciences, The University of Queensland, Brisbane, Queensland, Australia; 2 School of Chemistry and Molecular Biosciences, The University of Queensland, Brisbane, Queensland, Australia; 3 Department of Physiology, The University of Melbourne, Parkville, Victoria, Australia; Boston University School of Medicine, United States of America

## Abstract

Excessive exposure to alcohol prenatally has a myriad of detrimental effects on the health and well-being of the offspring. It is unknown whether chronic low-moderate exposure of alcohol prenatally has similar and lasting effects on the adult offspring’s health. Using our recently developed Sprague-Dawley rat model of 6% chronic prenatal ethanol exposure, this study aimed to determine if this modest level of exposure adversely affects glucose homeostasis in male and female offspring aged up to eight months. Plasma glucose concentrations were measured in late fetal and postnatal life. The pancreas of 30 day old offspring was analysed for β-cell mass. Glucose handling and insulin action was measured at four months using an intraperitoneal glucose tolerance test and insulin challenge, respectively. Body composition and metabolic gene expression were measured at eight months. Despite normoglycaemia in ethanol consuming dams, ethanol-exposed fetuses were hypoglycaemic at embryonic day 20. Ethanol-exposed offspring were normoglycaemic and normoinsulinaemic under basal fasting conditions and had normal pancreatic β-cell mass at postnatal day 30. However, during a glucose tolerance test, male ethanol-exposed offspring were hyperinsulinaemic with increased first phase insulin secretion. Female ethanol-exposed offspring displayed enhanced glucose clearance during an insulin challenge. Body composition and hepatic, muscle and adipose tissue metabolic gene expression levels at eight months were not altered by prenatal ethanol exposure. Low-moderate chronic prenatal ethanol exposure has subtle, sex specific effects on glucose homeostasis in the young adult rat. As aging is associated with glucose dysregulation, further studies will clarify the long lasting effects of prenatal ethanol exposure.

## Introduction

It is well recognised that an adverse intrauterine environment can have long lasting effects on the subsequent health of the adult. This concept is known as the Developmental Origins of Health and Disease (DOHaD) hypothesis. One clinical marker for an adverse intrauterine environment is low birth weight, a result of decreased fetal growth, which has been associated with characteristics of the metabolic syndrome including impaired glucose tolerance, insulin resistance and type 2 diabetes in adult life [1], [2], [3], [4], [5]. Animal models have demonstrated that intrauterine insults leading to alterations in glucose homeostasis include nutrient restriction [6], [7], prenatal glucocorticoid exposure [8], [9], [10], placental insufficiency [11], [12] and nicotine exposure [13]. In addition, both epidemiological studies and animal models indicate that males are more severely affected [14], [15]. The thrifty phenotype hypothesis suggests that poor fetal nutrition alters fetal metabolism to promote immediate survival [16]. This is achieved by increasing metabolic efficiency and storage of fuel sources in order to protect important tissues such as the brain, at the expense of others such as skeletal muscle and the endocrine pancreas [17]. These fetal adaptations are thought to remain in postnatal life where the altered metabolism of the offspring is incompatible with the increased nutrient supply, thereby placing the offspring at increased risk of obesity and adult onset type 2 diabetes.

In the last few decades it has become increasingly evident that prenatal alcohol (ethanol – EtOH) exposure may also alter glucose homeostasis and increase the risk of type 2 diabetes [18], [19]. Indeed, glucose intolerance and hyperinsulinaemia have been reported in children born with fetal alcohol syndrome [20]. In the fasted adult, alcohol has been shown to induce hypoglycaemia [21] through the interference of gluconeogenesis but with little effect on glycogenolysis [22]. Similarly, adult rats chronically fed EtOH develop hypoglycaemia, also due to decreased gluconeogenesis, with females being more severely affected than males [23]. In rats, fetal EtOH exposure has been shown to induce hypoglycaemia in late gestation as well as in early postnatal life [24], [25]. But unlike the adult, this fetal hypoglycaemia is associated with decreased hepatic glycogen stores [24], [26] and not decreased gluconeogenesis, as gluconeogenesis does not occur during fetal life for either rats or humans [27].

In rats, maternal ingestion of 25% weight/volume (w/v) EtOH (via the drinking water) for the entirety of pregnancy is associated with decreased fetal body and liver weights [25] as well as insulin resistance of offspring during the first three months of age, as indicated by elevated circulating insulin levels [18], [28]. Intra-gastric administration of EtOH (2 g/kg) to dams twice daily throughout gestation (a ‘binge’ model of maternal EtOH consumption) results in adult offspring with glucose intolerance [19], hyperglycaemia due to increased gluconeogenesis and hyperinsulinaemia [29], [30]. These studies indicate that prenatal EtOH exposure via the mother has long lasting effects on glucose homeostasis of the offspring, with different mechanisms to those seen following the direct consumption of EtOH by adults. However, these rodent studies involve maternal consumption of high concentrations of EtOH and not consumption at the low to moderate concentrations of alcohol that are reported to be consumed by many pregnant women [31], [32].

Recently we have developed a model of chronic low-moderate prenatal EtOH exposure whereby pregnant rats are fed a liquid diet containing 6% EtOH for the entirety of gestation [33]. This EtOH *ad libitum* feeding regimen produces a plasma EtOH concentration of ∼0.03% at 30 min after the onset of feeding, without causing maternal undernutrition or altering pregnancy outcomes (maternal weight gain, litter size, male to female ratio or gestational length). Using this model, the aim of the present study was to determine if chronic low-moderate prenatal EtOH exposure altered glucose homeostasis in rat offspring aged up to 8 months. We hypothesised that chronic low-moderate prenatal EtOH exposure induces sex specific alterations in glucose homeostasis that are less severe than those reported following high dose prenatal EtOH exposure [29], [34].

## Materials and Methods

### Animals

#### Ethics statement

This study was carried out in strict accordance with the recommendations in the Australian Code of Practice for the Care and Use of Animals for Scientific Purposes. The protocol was approved by the Animal Welfare Unit of The University of Queensland (animal ethics approval number: SBMS/304/10/NHMRC). All efforts were made to minimize suffering.

#### Dams

Treatment of dams to produce ethanol (EtOH)-exposed and control rats has been reported in detail previously [33]. Briefly, time-mated virgin Sprague-Dawley rats were housed in individual cages under controlled temperature and humidity with a 12 hour light (0000 h–200 h) – dark (1200 h–0000 h) light cycle. Dams were fed a liquid diet either lacking (Control, n = 39) or containing 6% (v/v) ethanol (EtOH, n = 38) from embryonic (E) day 1 until the pups were born at term. This amount of EtOH provided 15% EtOH-derived calories. The diets were made fresh daily and offered *ad libitum* for 21 hours per day at the onset of the dark cycle (midday), with water being provided for the remaining three hours per day. Upon spontaneous delivery of the pups, the liquid diet was removed and the dams provided a standard laboratory rat chow diet with water *ad libitum*. All dams nursed their own pups until weaning at postnatal (PN) day 28 at which time male and female offspring were separated and housed with same-sex littermates.

On day E8 (±2 days), 30 minutes after the fresh diet was provided, a mixed blood (venous and arterial) sample was collected from the tail of a subset of control and EtOH-fed dams (n = 7 per group). Whole blood glucose concentration was measured using an Accu-check Go glucometer (Roche Diagnostics, Castle Hill, NSW, Australia).

#### Fetal studies

A subset of dams (Control, n = 9; EtOH, n = 11) were killed at E20 as described previously [33] for the collection of fetal and maternal blood. Briefly, the anaesthetised (1 mL/kg i.p. ketamine/xylazine (50/50 mix of ketamine hydrochloride (100 mg/mL, Provet Pty. Ltd., Brisbane, Queensland, Australia) and Xylazil-20 (20 mg/mL, Provet Pty. Ltd., Brisbane, Queensland, Australia)) dam was placed supine and the abdomen and uterus opened to expose the fetuses. Fetal sex was determined and an incision made in the fetal chest wall overlying the heart for blood collection into a heparinised capillary tube. Blood from male and female fetuses of each litter was pooled to produce one male and one female blood sample per litter. Following removal of the fetuses a cardiac puncture was performed in the dam for the collection of maternal blood. Blood samples were centrifuged at 3,000×g for 10 min (4°C) for the determination of maternal and fetal plasma glucose concentrations.

#### Offspring studies

The day of delivery was defined as postnatal (PN) day 0. A subset of offspring (one to two males and females per litter) were killed at PN30 via cardiac puncture following anaesthesia (as described above) and pancreata collected and fixed whole in 10% buffered formalin for pancreatic histomorphology as described below. A second subset was fasted overnight and a mixed (arterial and venous) blood sample collected from the tail via tail slice [12] to measure circulating glucose and insulin concentration (at two, six and eight months) or triglyceride concentrations (at seven months; n = 11–15 male and female Control and EtOH-exposed offspring). A third subset of offspring underwent an intraperitoneal glucose tolerance test (IPGTT) and insulin challenge at four months of age (described below), with one recovery day between studies. At eight months of age, some animals underwent dual energy X-ray absorptiometry (DEXA) (n = 10 male and female Control and EtOH-exposed offspring) to determine body composition (lean mass, fat-free mass, fat mass and percentage fat mass [35]) while others were killed (1 mL/kg i.p. ketamine/xylazine) in the absence of fasting for collection of the liver, abdominal fat and gastrocnemius muscle for gene expression studies (detailed below).

#### Pancreatic histomorphometry

Formalin-fixed pancreata taken from PN30 male and female Control and EtOH-exposed offspring were processed from 10% buffered formalin to paraffin before being embedded whole in paraffin. Pancreata (n = 7–9 male and female Control and EtOH-exposed offspring) were sectioned exhaustively at 6 µm thickness using a Microm HM325 rotary microtome (Thermo Fischer Scientific, Wilmington, DE, USA), with sections collected every 200 µm. Sections (10–21 per animal) were mounted onto Superfrost microscope slides (Menzel-Glaser, Thermo Fischer Scientific, Wilmington, DE, USA) and stained with aldehyde fuchsin. Using point counting, the number of pancreatic islets and β-cells were estimated with light microscopy (Olympus BH2 light microscope, Olympus America Inc., Centre Valley, PA, USA) with a 20× objective. For each section, 25 fields of view were analysed by the same person, who was ‘blinded’ to the experimental group. The density of β-cells and islets was calculated as the ratio of β-cells/islets to pancreatic tissue and expressed as a percentage; β-cell/islet mass was determined as the product of density and pancreatic weight.

### In vivo Glucose Homeostasis Measures

#### Intraperitoneal glucose test (IPGTT) and insulin challenge

At 4 months of age offspring underwent both an IPGTT (n = 11–12 male and female Control and EtOH-exposed) and an insulin challenge (n = 11 male and female Control and EtOH-exposed) in a similar manner to that described previously [12]. Offspring were fasted overnight for the IPGTT and glucose (1 g/kg BW; Sigma-Aldrich, Sydney, NSW, Australia) administered i.p. Blood samples were collected from the tail, as indicated above, prior to glucose administration (−5 minutes) and at 5, 10, 20, 30, 45, 60 and 90 minutes after glucose administration. For the insulin challenge, rats received an i.p. injection of 0.75 U/kg body weight insulin (Actrapid, Novo Nordisk Pharmaceuticals Pty. Ltd., Baulkham Hills, NSW Australia) and tail blood samples were collected at −5, 20, 40, 60 and 90 and 120 minutes. Blood samples were centrifuged (3,000×g, 10 minutes, 4°C) and the plasma collected for determination of glucose (IPGTT and insulin challenge) and insulin (IPGTT) concentrations.

#### Plasma analyses

Glucose concentration was determined via colorimetric reaction using Infinity™ Glucose Oxidase Reagent (Thermo Scientific, Lidcomb, NSW, Australia) and measured at 500 nm (Cobas Mirra Chemistry Analyzer, Roche Diagnostics, Castle Hill, NSW, Australia). Plasma insulin was determined using a high sensitivity Rat Insulin Radioimmunoassay kit (Cat. # RI-13K, Millipore Australia Pty Ltd, Kilsyth, VIC, Australia; plasma samples diluted 1:2 with sample buffer) following the manufacturer supplied protocol. For insulin, intra- and inter-assay coefficients of variation were 3.2% and 9.2% respectively for a quality control of 2.9 ng/mL. Total area under the glucose curve and area under the insulin curve (total, first phase and second phase) curves were calculated using PRISM GraphPad PRISM 5 (GraphPad software, La Jolla, CA, USA). First phase insulin secretion was defined as the first 5 minutes after glucose administration with second phase insulin secretion being from 5 to 45 minutes [36]. The area under the total insulin and glucose curve ratio was calculated as an indicator of the insulin secretory response to glucose [12].

The homeostasis model assessment for insulin resistance (HOMA-IR) was calculated as published previously [37] and used as an indication of basal insulin sensitivity.

Triglyceride concentrations were analysed via colorimetric reaction (at 500 nm) with a Cobas Mirra Chemistry Analyzer (Roche Diagnostics, Castle Hill, NSW, Australia) and using Infinity™ Triglycerides reagents (Thermo Scientific, Lidcomb, NSW, Australia).

### Gene Expression Analysis

Relative gene expression was measured by real-time PCR in the liver, abdominal adipose tissue and gastrocnemius of offspring killed at 8 months of age (n = 6–12 male and female Control and EtOH-exposed offspring). Total RNA was extracted from tissues using an RNeasy Mini Kit (QIAGEN, Doncaster VIC, Australia), and reversed transcribed into cDNA using the Taqman reverse transcription reagents kit (Applied Biosystems, Scoresby, VIC, Australia). Real-time PCR was performed in the StepOne™ Real-Time PCR system (Applied Biosystems, VIC, Australia) and relative gene expression (in arbitrary units (AU)) analysed using the cycle of threshold fluorescence (Ct) method as reported previously [38]. Genes of interest, purchased as Assay on Demand (Applied Biosystems, Mulgrave, Vic, Australia; FAM-labelled probe), were: glucose transporter 2 (*GLUT2*; assay ID Mn00446224_m1), *GLUT4* (assay ID Rn00562597_m1), insulin receptor (*InsR*; assay ID Rn01403321_m1), forkhead box 01 (*Fox01*; assay ID Rn01494868_m1), peroxisome proliferator-activated receptor gamma, co-activator 1α (*PGC-1α*; assay ID Rn00580241_m1), phosphoenolpyruvate carboxykinase 1 (*PEPCK*; assay ID Rn01529014_m1), glucose-6-phosphate dehydrogenase (*G6Pase*; assay ID Rn00566576_m1), glucokinase (*GK*; assay ID Rn00561265_m1), *resistin* (assay ID Rn00595224_m1) and *adiponectin* (assay ID Rn00595250_m1). Each gene of interest was multiplexed with the house-keeping gene, 18s rRNA (VIC-labelled probe; TaqMan® Ribosomal RNA Control Reagents kit, Applied Biosystems, Mulgrave, VIC, Australia) as reported previously [33]. Thermo-cycling conditions during real-time PCR were as follows: enzyme activation at 50°C for 2 minutes and 95°C for 10 minutes followed by 40 cycles of denaturation for 15 seconds at 95°C and annealing plus extension for 60 seconds at 60°C.

### Statistical Analyses

For all statistical analyses, comparisons were made between Control and EtOH-exposed offspring of the same sex using the statistical package SigmaStat (Systat Software, San Jose, CA, USA). Means were compared using a two-tailed unpaired Students t-test or, in the case of a failed normality test, a Mann-Whitney Rank Sum test, or a two-way ANOVA with prenatal exposure and age as factors and a Tukey *post hoc* analysis. Values are expressed as mean±SEM. The level of significance was taken as P<0.05.

## Results

### Maternal and Fetal Measurements

Maternal parameters and gestational outcome have been reported previously [33]. Briefly, there was no difference in maternal weight gain or dietary consumption, gestational length, litter size or ratio of male to female offspring between Control and EtOH-fed dams. At PN1 when the offspring were first weighed, there were no differences in body weight between groups (male control: 7.07±0.30 g, EtOH-exposed: 6.80±0.28 g, female control: 6.81±0.22 g, female EtOH-exposed: 6.63±0.23 g).

**Figure 1:**
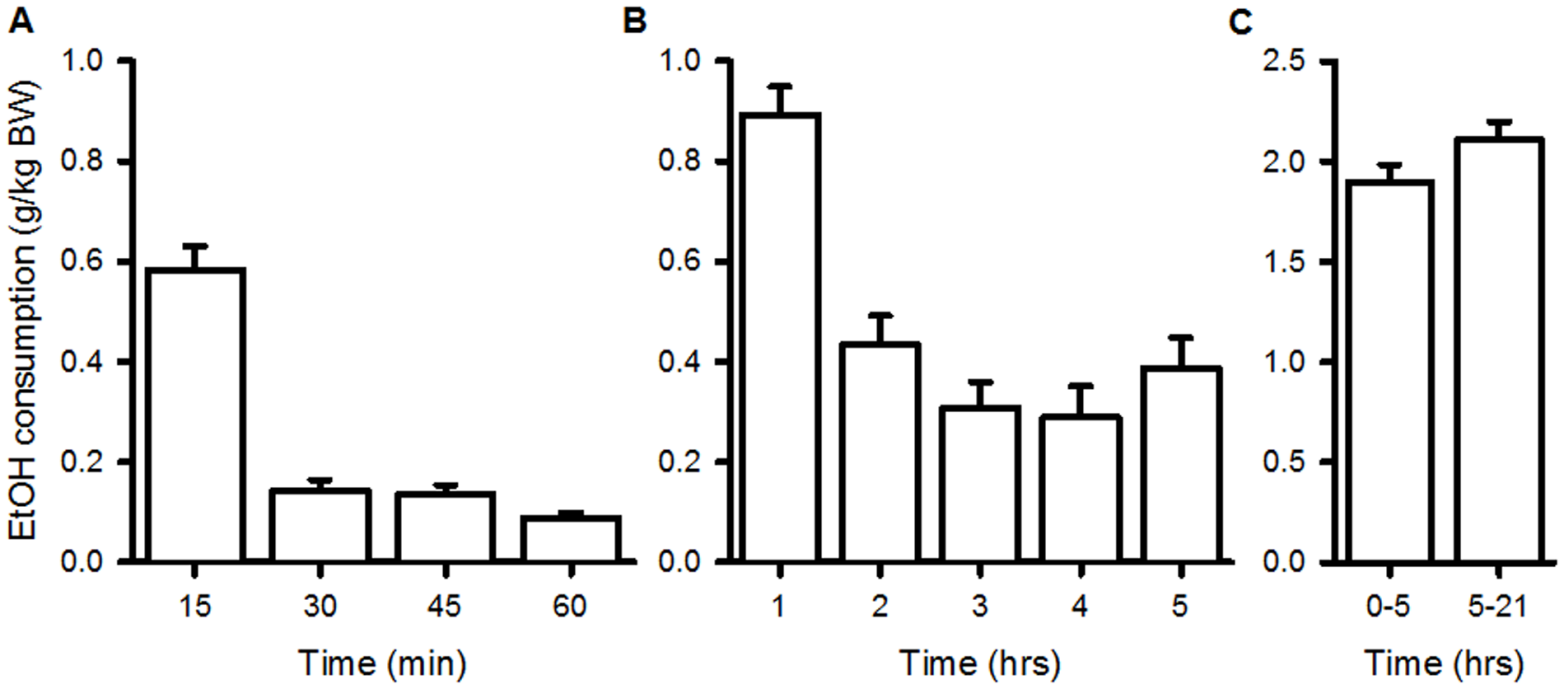
Daily pattern of ethanol (EtOH) consumption by pregnant dams. The average amount of EtOH consumed within each (A) 15 minute interval during the first hour (n = 5), (B) one hour interval during the first 5 hours (n = 4) and (C) from 0–5 hours and 5–21 hours (n = 38). Data are expressed as mean of mean±SEM.

The amount of EtOH consumed (g/kg BW) within each period of dietary consumption recorded is shown in Figure 1. The average hourly consumption of EtOH was 0.21±0.01 g/kg/hr. This produced a measured plasma EtOH concentration of 0.03% [33].

**Figure 2:**
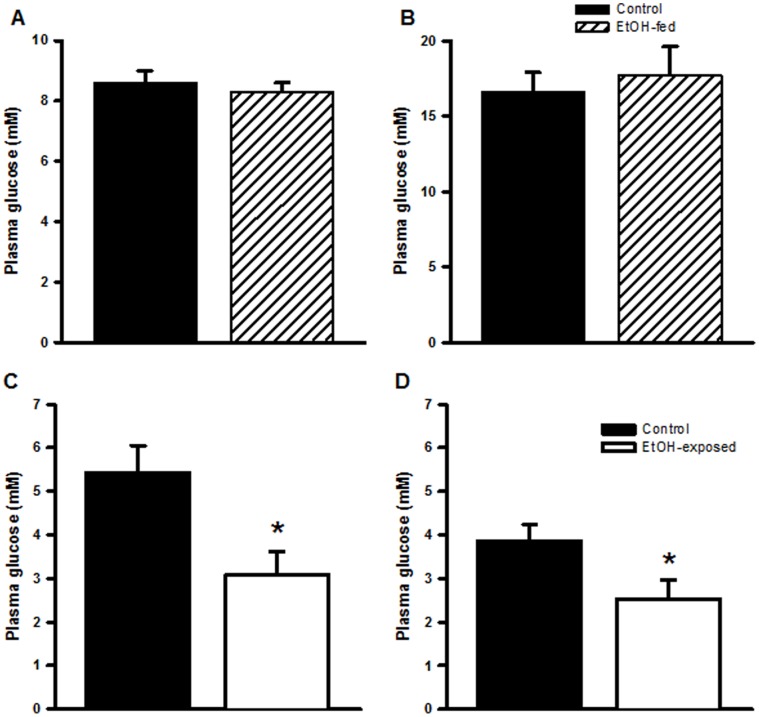
Maternal and fetal glucose concentration during ethanol consumption/exposure. Maternal plasma glucose concentration at (A) at embryonic day (E) 8 and (B) E20, and fetal plasma glucose concentration of (C) male and (D) female fetuses at E20. Data are expressed as mean±SEM. *P<0.05.

At both E8 and E20 there were no differences in plasma glucose concentrations between Control and EtOH-fed dams (Figure 2A and B). In contrast, at E20 EtOH-exposed fetuses had a significantly lower plasma glucose concentration than their control counterparts (males P = 0.012, females P = 0.04; Figure 2C and D).

### Pancreatic Histomorphometry

For the subset of offspring killed at PN30, there was no difference in body weight, pancreas weight or pancreas weight relative to body weight within male or female offspring groups (Table 1). Similarly, there was also no difference between Control and EtOH-exposed male or female offspring in β-cell density, islet density, β-cell mass or islet mass (Table 1).

**Table 1:** Body and pancreas weight, and pancreatic histomorphology of Control and prenatal ethanol (EtOH)-exposed rats at 30 days of age.

	Male		Female	
	Control	EtOH-exposed	Control	EtOH-exposed
Body wt (g)	88±3	85±4	77±3	73±3
Absolute pancreas wt (mg)	352±12	372±24	358±30	314±17
Relative pancreas wt (mg/g BW)	4.04±0.17	4.40±0.22	4.65±0.26	4.31±0.15
β-cell density (%)	0.96±0.23	0.97±0.22	1.35±0.20	1.55±0.21
Islet density (%)	1.20±0.26	1.27±0.26	1.62±0.25	1.86±0.25
β-cell mass (mg)	3.42±0.81	3.50±0.67	4.78±0.89	4.94±0.75
Islet mass (mg)	4.24±0.90	4.66±0.84	5.74±1.08	5.89±0.91

Offspring were prenatally exposed to a control (Control) or 6% EtOH containing diet for the entirety of gestation and pancreatic histomorphology determined at postnatal day 30 (n = 8–9 male and female Control and EtOH-exposed). A Students t-test was used to compare groups within each sex. Data are expressed as mean±SEM. wt – weight; BW – body wt.

### Fasting Parameters

#### Plasma glucose and insulin concentrations

**Figure 3:**
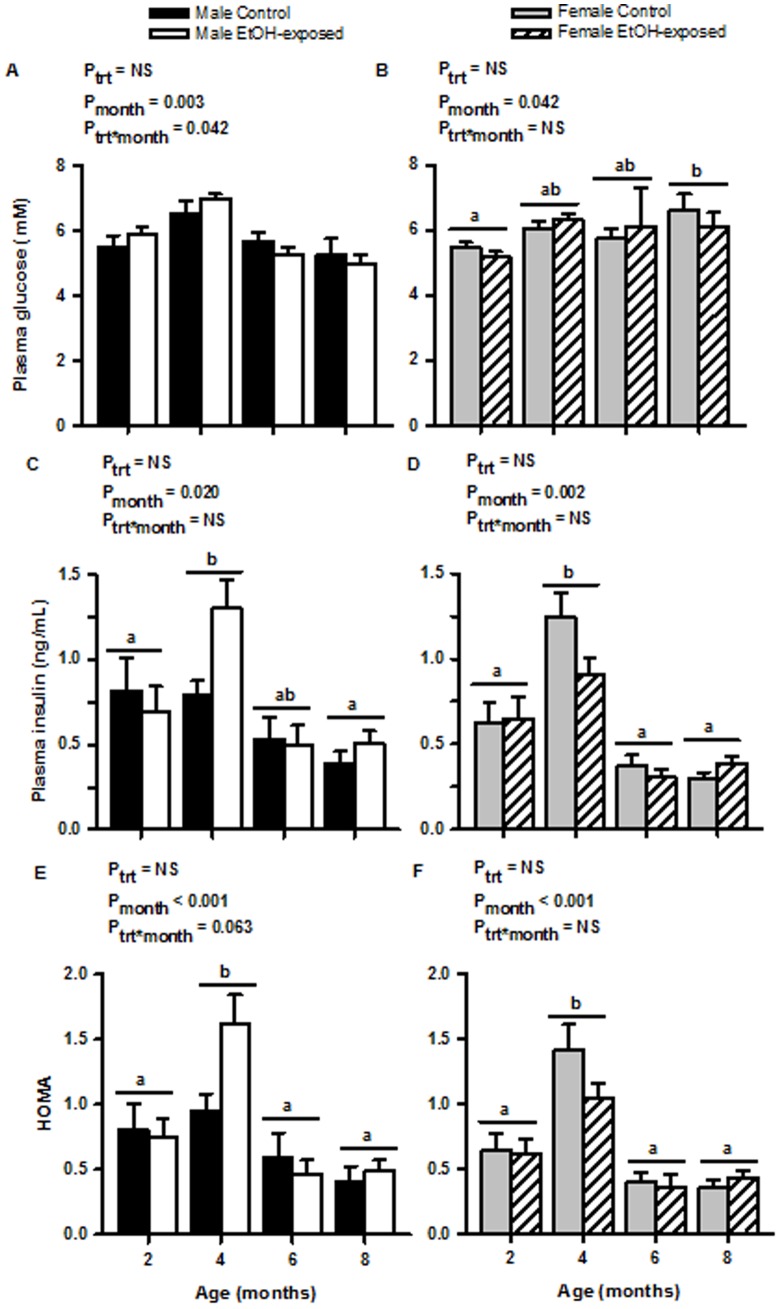
Fasting plasma glucose and insulin concentrations and homeostasis model assessment for insulin resistance (HOMA-IR). Fasting plasma (A, B) glucose concentration, (C, D) insulin concentration and (E, F) the associated HOMA-IR of male (A, C, E) and female (B, D, F) Control and EtOH-exposed offspring at 2, 4, 6 and 8 months of age. Data are expressed as mean±SEM. Ptrt, Pmonth and Ptrt*month refer to the ANOVA p-values regarding the effects of treatment, month and the interaction between treatment and month, respectively. Different letters indicate significant differences across the ages (‘a’ is different to ‘b’ but ‘ab’ is not different to ‘a’ or ‘b’).

Longitudinal analyses of plasma glucose (Figure 3A and B) and insulin (Figure 3C and D) concentrations during the first eight months of postnatal life did not reveal any differences between male or female Control and EtOH-exposed offspring. For plasma glucose concentrations in males, despite the original ANOVA indicating a change over time and an interaction between treatment and time, the post-hoc analysis did not reveal any differences. However, for males both plasma insulin and HOMA-IR were increased at four months. For females, plasma glucose concentrations increased with age and plasma insulin and HOMA-IR were increased at four months. (Figure 3E and F).

#### Plasma triglycerides

At seven months of age triglyceride levels were 18% lower (P = 0.105) in male EtOH-exposed offspring (0.71±0.05 mM) than their control counterparts (0.87±0.07 mM). In contrast, plasma triglyceride levels were similar for female control and EtOH-exposed offspring (0.69±0.05 mM versus 0.64±0.04 mM).

### Intraperitoneal Glucose Tolerance Test

**Figure 4:**
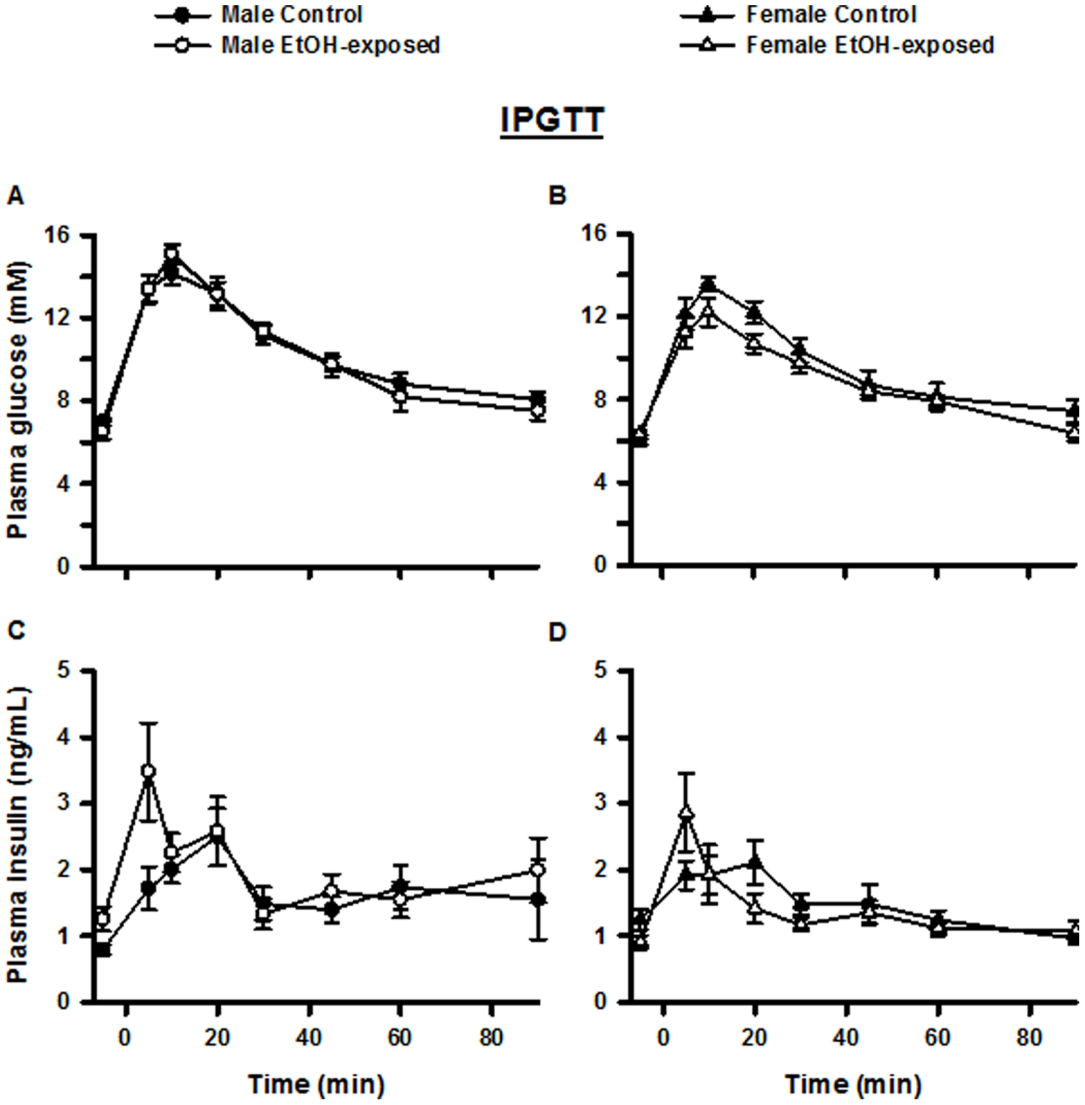
Plasma glucose and insulin response curves during the glucose tolerance test and insulin challenge. Response curves during the (A, B, C, D) intraperitoneal glucose tolerance test (IPGTT) and (E, F) insulin challenge. Plasma glucose (A, B) and insulin (C, D) concentrations during the IPGTT and plasma glucose concentrations (E, F) during the insulin challenge are shown for male (A, C, E) and female (B, D, F) Control and EtOH-exposed offspring. Data are expressed as mean±SEM.

**Figure 5:**
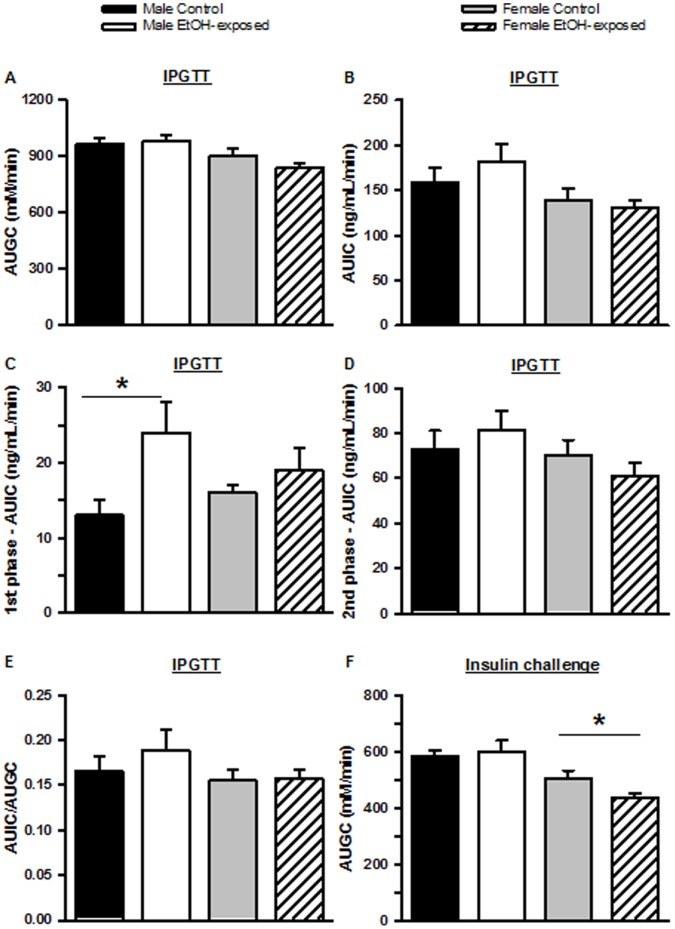
Area under the curve during the glucose tolerance test and insulin challenge. (A) Area under the glucose curve (AUGC) and area under the insulin curve (AUIC) for (B) total insulin secretion, (C) 1^st^ phase insulin secretion and (D) 2^nd^ phase insulin secretion as well as (E) ratio of AUIC to AUGC during the intraperitoneal glucose tolerance test (IPGTT). (F) AUGC during the insulin challenge. Data are expressed as mean±SEM. *P<0.05 compared to same sex control.

The response to i.p. glucose administration is shown in Figures 4A–D. At the time of the IPGTT male Control and EtOH-exposed offspring weighed 405±12 g and 411±12 g, respectively (not different), while female Control and EtOH-exposed offspring weighed 242±6 g and 247±5 g, respectively (not different). Basal concentrations of glucose were similar within male (6.5±0.4 mM versus 7.0±0.1 mM) and female (6.0±0.2 mM versus 6.3±0.2 mM) groups. In contrast, basal insulin concentrations were significantly increased in male EtOH-exposed offspring (0.8±0.1 ng/mL versus 1.3±0.2 ng/mL; P = 0.029) and decreased, though not significantly so, in female EtOH-exposed offspring (1.2±0.1 ng/mL versus 0.9±0.1 ng/mL; P = 0.065). During the IPGTT the area under the glucose curve was not different between male or female Control and EtOH-exposed offspring (Figure 5A). Similarly, the total area under the insulin curve was not different between male or female Control and EtOH-exposed groups (Figure 5B). However, when first (0 to 5 minutes) and second (5 to 45 minutes) phase insulin secretions were analysed, it was found that male, but not female, EtOH-exposed offspring had a greater (P = 0.009) first phase (Figure 5C), but not second phase (Figure 5D), insulin secretion than Controls. There ratio of area under the insulin curve to area under glucose curve was not different between male and female groups (Figure 5E).

### Insulin Challenge

The response to i.p. insulin administration is presented in Figures 4E and F. At the time of the insulin challenge, male Control and EtOH-exposed offspring weighed 425±10 g and 415±11 g, respectively (not different), and female Control and EtOH-exposed offspring weighed 256±6 g and 252±5 g, respectively (not different). Basal plasma glucose concentrations were 7.7±0.2 mM, 7.8±0.2 mM, 7.4±0.2 mM and 7.6±0.1 mM for male Control, male EtOH-exposed, female Control and female EtOH-exposed offspring, respectively. Although there was no difference in the area under the glucose curve between male Control and EtOH-exposed offspring, the area under the glucose curve was significantly less (P = 0.044) for female EtOH-exposed offspring compared to controls (Figure 5F).

### Body Composition

Lean muscle mass at eight months of age was not different between male Control and EtOH-exposed offspring or female Control and EtOH-exposed offspring. Similarly, there was no difference between male or female groups in fat-free mass, fat mass, per cent body fat or total body mass (Table 2).

**Table 2:** Body composition of eight month old Control and prenatal ethanol (EtOH)-exposed rats.

	Male		Female	
	Control	EtOH-exposed	Control	EtOH-exposed
Lean mass (g)	407±17	378±15	232±8	233±8
Fat free mass (g)	423±18	393±15	241±8	243±8
Fat mass (g)	119±8	133±14	53±6	51±5
% fat (g)	22±1	25±2	18±2	17±2
Total body mass (g)	542±19	526±17	294±9	294±8

Male and female Control and prenatal EtOH-exposed rats (n = 10 per group) underwent whole body composition analyses using dual energy X-ray absorptiometry. A Students t-test was used to compare groups within each sex. Data are expressed as mean±SEM.

### Gene Expression

Prenatal EtOH exposure did not alter the expression of metabolic genes within the abdominal adipose tissue, gastrocnemius muscle or liver (Table 3).

**Table 3:** Relative hepatic, abdominal adipose and gastrocnemius gene expression of eight month old control and prenatal ethanol (EtOH)-exposed rats.

	Male		Female	
	Control	EtOH-exposed	Control	EtOH-exposed
Liver				
GLUT2	0.90 [0.40, 3.49]	0.40 [0.26, 1.44]	0.93 [0.66, 1.23]	0.99 [0.72, 1.72]
GLUT4	0.86 [0.76, 1.35]	0.90 [0.52, 1.14]	1.14±0.20	1.49±0.31
PEPCK	1.22±0.14	1.29±0.21	1.21 [1.00, 1.39]	1.19 [0.98, 1.79]
PGC-1α	1.37±0.31	1.68±0.74	0.99 [0.54, 1.76]	0.88 [0.69, 1.20]
G6Pase	1.22±0.20	0.83±0.17	1.16±0.16	1.14±0.23
GK	1.13±0.18	0.86±0.29	1.30±0.21	1.29±0.22
InsR	1.27±0.28	0.99±0.19	1.00 [0.51, 1.76]	0.61 [0.50, 1.03]
FoxO1	1.08 [0.51, 1.54]	0.75 [0.60, 3.50]	1.42±0.47	1.84±0.59
Adipose tissue				
Resistin	1.17±0.21	1.24±0.30	1.42±0.30	1.27±0.17
Adiponectin	1.02±0.07	0.80±0.14	1.05±0.10	1.10±0.07
GLUT4	1.31±0.30	1.27±0.29	1.29±0.26	1.74±0.25
InsR	1.06±0.11	0.90±0.17	1.09±0.13	1.23±0.08
Gastrocnemius				
PGC-1α	1.38±0.38	1.05±0.27	1.24±0.26	0.91±0.14
GLUT4	1.09±0.12	1.05±0.22	1.15±0.21	0.93±0.16

Rats were prenatally exposed to a control or 6% EtOH containing diet for the entirety of gestation. Group sizes were n = 6–12 per group. Data are expressed as mean±SEM (comparison of data from same-sex groups via Students t-test) or median [25%, 75% confidence intervals] (comparison of data from same-sex groups via Mann-Whitney Rank Sum test). GLUT – glucose transporter; PEPCK – phosphoenolpyruvate carboxykinase 1; PGC-1α – peroxisome proliferator-activated receptor gamma, coactivator 1α; G6Pase – glucose-6-pohosphotase; GK – glucokinase; InsR – insulin receptor; FoxO1– forkhead box protein 1.

## Discussion

Using a rodent model of chronic low-moderate dose maternal EtOH consumption, we have shown that this type of fetal EtOH exposure induces fetal hypoglycaemia that is independent of maternal glucose status. In addition, prenatal EtOH exposure has differential effects on glucose homeostasis of male and female offspring. Although pancreatic histomorphometry at PN30 was not significantly altered by low-moderate chronic prenatal EtOH exposure, four month old male EtOH-exposed offspring were hyperinsulinaemic and displayed elevated first phase insulin secretion in a glucose challenge, suggesting some degree of insulin resistance. In contrast, at this same age female EtOH-exposed offspring appeared to be hypoinsulinaemic under basal conditions and had a lower area under the glucose curve during the insulin challenge, suggesting increased insulin sensitivity. These results indicate that chronic low-moderate dose prenatal EtOH exposure may alter the programming of glucose homeostasis and induce metabolic abnormalities in postnatal life in a sex specific manner.

As EtOH is known to induce hypoglycaemia in both humans [21] and rats [26], it was not surprising that the EtOH-exposed fetuses were hypoglycaemic. We have previously reported these same fetuses are modestly growth restricted [33] consistent with reduced fetal nutrition. Fetal nutrition has been shown previously to be important for pancreatic development and function. Epidemiological studies demonstrate that adult onset glucose intolerance or insulin resistance of offspring may be the result of maternal undernutrition during pregnancy [1], [39]. In rodents, a low-protein maternal diet for the entirety of gestation decreases pancreatic islet size in offspring at birth [40] and maternal food restriction during late gestation decreases β-cell mass in the neonate [41], [42]. Maternal undernutrition affects the delivery of a number of macro and micro-nutrients to the fetus, including glucose. Glucose is the major stimulus of β-cell replication and stimulates the entry of β-cells into an active cell cycle (reviewed in [43]). Fetal hypoglycaemia may therefore prevent pancreatic development, leading to decreased β-cell mass and lowering insulin production and secretion postnatally. Hence, we conducted our study of β-cell mass at PN 30 to assess if the offspring have a pancreatic deficit from early postnatal life.

In this study, it was expected that EtOH exposed offspring would have abnormal or delayed pancreatic development, however this was not observed at PN30. It is possible that fetuses in our study were only acutely hypoglycaemic at the time of measurement in late gestation, a period that coincides with both rapid fetal growth [44] and increases in β-cell mass [45], and not for the entirety of gestation. If this were the case, it would suggest that pancreatic development is not disturbed by chronic exposure to low-moderate levels of EtOH. Alternatively, as pancreatic development continues postnatally [46] and β-cells are known to self-replicate [47], it could be that the removal of EtOH at birth allowed normoglycaemia to be achieved resulting in accelerated pancreatic development and maturation in the young EtOH-exposed offspring such that differences were not observed at PN30. Furthermore, body weights and growth profiles of EtOH exposed offspring in this model were normal up to 7 months of age [33], highlighting that prenatal growth restriction can be overcome upon removal of the EtOH-exposure at birth. Such a phenomenon (the postnatal restoration of β-cell mass following the removal of the fetal insult with birth) has been demonstrated previously in a rodent model of uteroplacental insufficiency that was followed by cross-fostering at birth to improve postnatal nutrition [48]. Thus, the lactational environment may be a critical developmental stage for the programming of adult disease and may act as a modifier for the impact of prenatal challenges. Our findings of normoglycaemia and normoinsulinemia at two months of age following modest prenatal EtOH exposure indicates this latter suggestion may be the case. Although chronic low-moderate prenatal EtOH exposure did not appear to alter pancreatic development in our model, it is possible that the present fetal insult may accelerate the normal decrease in β-cell mass that is seen with senescence, as observed in male rats that experienced intrauterine undernutrition [49]. Thus, it would be of value to assess in future studies whether prenatal EtOH exposure accelerates this process and leads to decreased β-cell mass with age.

Although no difference was seen between Control and EtOH-exposed groups in fasting basal glucose and insulin concentrations during the longitudinal analyses, basal glucose concentrations increased with age in females but not males. For both sexes, basal plasma insulin concentrations (and HOMA-IR) were elevated at four months compared to the other ages. It must be remembered that plasma insulin concentration at four months of age was determined from the basal blood sample collected during the GTT. Therefore we do not believe the increased insulin concentration is of biological significance to glucose homeostasis *per se*, but suggest it reflects a stress response to the unique housing conditions for the GTT. This hypothesis could be confirmed in future experiments by measuring circulating cortisol concentrations. Also observed at this age were subtle alterations in glucose clearance, glucose tolerance and insulin resistance when glucose homeostasis was challenged through the glucose tolerance test and insulin challenge. These alterations were sexually dimorphic with male EtOH-exposed offspring having some insulin resistance (as indicated by an elevated HOMA index under basal conditions and elevated first phase insulin secretion) and female EtOH-exposed offspring having increased insulin sensitivity. Such sexual dimorphism has also been reported in Wistar rats chronically fed a high dose EtOH diet postnatally [23] and in adult sheep that were born of ewes that were malnourished from 61 days prior to and 30 days following conception [10]. Interestingly, in the latter study female offspring of undernourished mothers also demonstrated enhanced glucose clearance [10]. Such sex-specific alterations in postnatal physiology following prenatal insults have observed with many models (for review see [50]).

The changes observed in the present study were similar, yet more subtle, than the overt changes seen in offspring following high dose prenatal EtOH exposure [19], [29], [30], [51]. In those studies, where pregnant rats were administered 2 g/kg EtOH twice daily via gavage, it was found that male EtOH-exposed offspring were insulin resistant and glucose intolerant at three and four months of age. Together, both our study and those of Chen and Nyomba demonstrate that prenatal EtOH exposure induces insulin resistance in male offspring [19], [29]. Insulin secretion is a two phase process with the first phase involving the rapid secretion of stored insulin over an 8 minute period, and the second phase involving the slow secretion of predominantly newly synthesised insulin [36]. Male EtOH-exposed offspring of the present study displayed increased first phase insulin secretion, indicating large insulin stores within the pancreas, and normal second phase insulin secretion, indicating pancreatic β-cells were able to adequately synthesis and secrete insulin. However, the hypersecretion of insulin during the first phase did not result in a significantly reduced plasma glucose concentration. Whether this was due to central or peripheral insulin resistance or elevated gluconeogenesis at this time is unknown. Using their acute model of high dose prenatal EtOH exposure, Chen and Nyomba showed that 12 week old EtOH-exposed, insulin resistant male offspring had normal gastrocnemius GLUT4 protein levels in the fed state but decreased levels in when in the fasted state [19] indicating peripheral insulin resistance during fasting. In another study, this same group found that 14 week old insulin resistant male offspring had elevated PEPCK activity as well as increased hepatic PEPCK and PGC-1α mRNA and protein levels following a pyruvate challenge [34]. From this the authors concluded that the glucose intolerance was due to a lack of inhibition of gluconeogenesis following insulin administration. Based on these findings, it is possible that male EtOH-exposed offspring of the present study may have had either increased gluconeogenesis or peripheral insulin resistance, however in the absence of performing biochemical studies on either fasted EtOH-exposed offspring of the same age or EtOH-exposed offspring at the conclusion of first phase insulin secretion during the insulin challenge we are unable to determine the molecular mechanisms of this insulin resistance. It is possible that the male EtOH-exposed offspring in the current study were hypoleptinaemic as hypoleptinaemia in humans and mice has been associated with insulin resistance, hyperinsulinaemia, hyperglycaemia and hypertriglyceridaemia (reviewed in [52]). Although we were unable to collect sufficient blood to determine circulating leptin levels in the current study, this would be of interest in the future.

At first glance it appears in the present study that chronic low-moderate level prenatal EtOH exposure in females may be beneficial (due to the enhanced glucose clearance observed) but this may in fact be detrimental to the long term health of the offspring; improved glucose clearance may lead to increased conversion of glucose into triglycerides and increased fat deposition. There are strong links between poor fetal nutrition (indicated by low birth weight), elevated triglycerides postnatally and, ultimately, obesity in males and females that can be detected as early as in childhood (reviewed in [53]). However, the association between an adverse fetal environment, obesity and glucose metabolism appears to be more dependent upon total body fat content than body mass index (reviewed in [54]). Subsequent to our findings of enhanced glucose clearance in female EtOH-exposed offspring at 4 months, we explored this association between an adverse intrauterine environment, enhanced glucose clearance, elevated triglyceride concentrations and body composition. We then killed animals for collection of abdominal adipose tissue, thereby allowing measurement of resistin and adiponectin gene expression. We found that EtOH-exposed offspring had normal plasma triglyceride concentrations and fat deposition. Although resistin (a marker of adiposity), and adiponectin have been linked to obesity and diabetes [55], [56], these genes were unaltered by chronic low-moderate prenatal EtOH exposure. In contrast, high dose prenatal EtOH exposure elevates adipose resistin mRNA as well as circulating plasma resistin levels in rats and, when fed a high fat diet to simulate a nutritionally plentiful postnatal environment, heavier epididymal fat pads [51]. Together, these results indicate that offspring of the present study may not be tending towards obesity-associated abnormal glucose homeostasis.

Gene expression levels were measured in the liver, gastrocnemius muscle and abdominal adipose tissue of eight month old animals in an attempt to detect central and/or peripheral insulin resistance. Despite exploring genes involved in glucose clearance, glucose breakdown and gluconeogenesis, we did not detect differences in gene expression of male or female EtOH-exposed offspring. As the lack of gene changes concurred with the normoglycaemic and normoinsulinaemic status of EtOH-exposed offspring during monthly fasting conditions, we had no evidence to suggest regulation of glucose homeostatic feedback pathways at the protein level and therefore we did not measure protein expression. In contrast, our gene expression results do not concur with the alterations in insulin secretion or glucose tolerance that were observed in male EtOH-exposed offspring during the glucose tolerance test. Thus, we were unable to reveal any molecular mechanisms. Previously Chen *et. al.* found that *GLUT4* mRNA levels within the gastrocnemius were not altered by high-dose prenatal EtOH exposure when rats were in the fed state [19]. However, when in the fasted state high-dose EtOH-exposed rats displayed differential regulation for *GLUT4* mRNA within the gastrocnemius [19]. Furthermore, in the Chen study the gastrocnemius appeared to be collected from rats following an IPGTT. Therefore, in our current study the disparity between appropriately regulated gene expression of fed rats and abnormal insulin secretion and glucose tolerance during the glucose tolerance test given to fasted rats may be due to inappropriate tissue collection; collection of tissue from fasted animals and/or immediately following the glucose tolerance test may have revealed the molecular mechanisms.

In conclusion, this is the first study to explore the effect of chronic low-moderate prenatal EtOH exposure on glucose homeostasis in rats. Whilst this treatment did not cause changes in basal glucose and insulin concentrations, subtle differential alterations were seen in EtOH-exposed offspring during challenges, with four month old males displaying insulin resistance and females displaying enhanced glucose clearance. Further longitudinal studies need to be performed using aged rats to determine if this chronic, low-moderate prenatal insult induces more overt alterations in glucose homeostasis with age. Despite this, results of the current study suggest even modest amounts of alcohol during pregnancy can induce long term alterations in glucose regulation in offspring, thereby highlighting the need for abstinence in pregnant women.
